# Engineered bone marrow mesenchymal stem cell-derived exosomes loaded with miR302 through the cardiomyocyte specific peptide can reduce myocardial ischemia and reperfusion (I/R) injury

**DOI:** 10.1186/s12967-024-04981-7

**Published:** 2024-02-17

**Authors:** Jianjun Gu, Jia You, Hao Liang, Jiacai Zhan, Xiang Gu, Ye Zhu

**Affiliations:** 1 Department of Cardiology, Institute of Translational Medicine, Medical College, Yangzhou University, Yangzhou, Jiangsu China; 2 Department of Cardiology, Northern Jiangsu People’s Hospital, 98 Nantong West Road, Yangzhou, Jiangsu China; 3 Department of Internal Medicine, Yangzhou Maternal and Child Health Care Hospital, Yangzhou, 225001 Jiangsu China

**Keywords:** Exosome, Gene delivery, miR302, BMSCs, Myocardial infarction

## Abstract

**Background:**

MicroRNA (miRNA)-based therapies have shown great potential in myocardial repair following myocardial infarction (MI). MicroRNA-302 (miR302) has been reported to exert a protective effect on MI. However, miRNAs are easily degraded and ineffective in penetrating cells, which limit their clinical applications. Exosomes, which are small bioactive molecules, have been considered as an ideal vehicle for miRNAs delivery due to their cell penetration, low immunogenicity and excellent stability potential. Herein, we explored cardiomyocyte-targeting exosomes as vehicles for delivery of miR302 into cardiomyocyte to potentially treat MI.

**Methods:**

To generate an efficient exosomal delivery system that can target cardiomyocytes, we engineered exosomes with cardiomyocyte specific peptide (CMP, WLSEAGPVVTVRALRGTGSW). Afterwards, the engineered exosomes were characterized and identified using transmission electron microscope (TEM) and Nanoparticle Tracking Analysis (NTA). Later on, the miR302 mimics were loaded into the engineered exosomes via electroporation technique. Subsequently, the effect of the engineered exosomes on myocardial ischemia and reperfusion (I/R) injury was evaluated in vitro and in vivo, including MTT, ELISA, real-time quantitative polymerase chain reaction (PCR), western blot, TUNNEL staining, echocardiogram and hematoxylin and eosin (HE) staining**.**

**Results:**

Results of in vitro experimentation showed that DSPE-PEG-CMP-EXO could be more efficiently internalized by H9C2 cells than unmodified exosomes (blank‐exosomes). Importantly, compared with the DSPE-PEG**-**CMP-EXO group, DSPE-PEG**-**CMP-miR302-EXO significantly upregulated the expression of miR302, while exosomes loaded with miR302 could enhance proliferation of H9C2 cells. Western blot results showed that the DSPE-PEG**-**CMP-miR302-EXO significantly increased the protein level of Ki67 and Yap, which suggests that DSPE-PEG-CMP-miR302-EXO enhanced the activity of Yap, the principal downstream effector of Hippo pathway. In vivo, DSPE-PEG**-**CMP-miR302-EXO improved cardiac function, attenuated myocardial apoptosis and inflammatory response, as well as reduced infarct size significantly.

**Conclusion:**

In conclusion, our findings suggest that CMP-engineered exosomes loaded with miR302 was internalized by H9C2 cells, an in vitro model for cardiomyocytes coupled with potential enhancement of the therapeutic effects on myocardial I/R injury.

## Introduction

Myocardial infarction (MI) is the leading cause of morbidity and mortality worldwide [1]. Once MI occurs, cardiomyocytes are irreversibly damaged, which leads to decreased cardiac function, myocardial remodeling, and ultimately heart failure (HF) [2]. Despite percutaneous coronary intervention (PCI) and coronary artery bypass grafting have been considered as effective strategies for MI, patients with MI still experience significant mortality [3]. Therefore, new effective treatments are needed to protect the myocardium from ischemia and reperfusion (I/R) injury. Previous studies have shown that certain miRNAs are beneficial for improving cardiac function after MI [4–6]. In addition, other studies have confirmed that some miRNAs could induce cardiomyocyte proliferation [5, 7]. In particular, it has been reported that miRNA302 promoted cardiomyocyte proliferation and regeneration after MI through the inhibition of Hippo pathway [8]. Yes-associated protein (Yap), the transcriptional effector of the Hippo pathway is translocated to the nucleus to interact with the transcriptional enhancer factor domain family for the activation of cellular proliferation related genes [9]. In particular, some studies have demonstrated that the proliferation of cardiomyocytes may potentially facilitate treatment of myocardial I/R injury. Particularly, Maring et al. observed an increased level of Yap (a Ki67-associated protein, another marker of cell proliferation), a marker of cardiomyocytes proliferation in the whole infarcted area [10]. In another study, it was suggested that the cell cycle activity of cardiomyocytes needs Yap, which is the principal downstream effector of Hippo pathway [11]. Besides, Lin et al. observed that activation of Yap resulted in attenuation of myocardial injury, and enhanced the function of cardiomyocytes [12]. Of note, the clinical application of miRNA-based therapy still faces numerous challenges, wherein scientists have sought to improve the delivery of miRNAs without degradation, retention at the infarct site, and efficient entry into target cells.

Exosomes are nano-sized membrane vesicles with their diameter ranging from 40 to 100 nm [13], wherein they can transfer small molecules (protein, miRNAs, mRNAs and lipids) to facilitate cell-to-cell communication [14]. In view of their low immunogenicity, high stability and permeability to biological barrier, exosomes have been considered as ideal carrier for delivery of therapeutic miRNA [15]. For example, Lv et al. reported that loading of miR21-5p into human adipose stem cell derived exosomes could further promote diabetic cutaneous wound healing [16]. Also, Yuan et al. found that exosomes loaded with miRNA29b prevented cardiac fibrosis after MI [17]. Nevertheless, practical applications of exosomes remain challenging due to their off-targeting, low capacity of specific miRNAs and short time retention in vivo [18–20]. Therefore, innovative approaches are required to deliver, target and retain the exosomes to the lesion site to promote tissue repair.

In this study, we developed a targeted peptide-modified exosome for the sustained release of miRNA302. To achieve this aim, we first modified a CMP with 1,2-distearoyl-*sn*-glycero-3-phosphoethanolamine-*N*-[hydroxysuccinimidyl (polyethylene glycol)-2000] (DSPE-PEG-NHS), then the PEG-modified protein peptide was ligated to the exosome. Subsequently, miR302 mimics were loaded into engineered exosomes via electroporation technique. Engineered exosomes demonstrated improved exosomal uptake into cardiomyocytes, promoted cardiomyocyte proliferation in vitro, and decreased myocardial apoptosis and inflammatory response, improved cardiac function in vivo. These findings laid the foundation for the application of engineered exosomes enriched with miR302 for the clinical treatment of MI.

## Methods

### BMSCs isolation and culture

Bone mesenchymal stem cells (BMSCs) were extracted from C57BL/6 mice (2–3 weeks old) via whole bone marrow adherence method. Briefly, we obtained the tibia and femur after the mice have been anesthetized by ketamine (80 mg/kg) and xylazine (5 mg/kg). Later on, the bones were cut from both ends, while the bone marrow cells were flushed with a syringe. Subsequently, the obtained suspension was filtered with a 70-μm cell strainer, before centrifugation was carried out at 500 g and 4 °C for 5 min. Ultimately, the cells were cultured in DMEM with 10% fetal bovine serum (FBS) and 1% penicillin–streptomycin in a 10-cm culture dish containing 5% CO_2_ at 37 °C. The BMSCs from the third passage were applied for the following experiments.

### BMSCs identification

BMSCs were digested with trypsin solution, washed by PBS, resuspended with serum-containing PBS buffer, and aliquoted into EP tubes (200 μL per tube). The cells were incubated with CD34, CD45, CD29, and CD90 antibodies (Abclonal, China) for 30 min in the dark at 4 °C. After incubation, the cells were detected with a FACS Calibur II flow cytometer (BD Biosciences) and analyzed via Flow Jo software (Flow Jo, USA).

Moreover, the specific markers (CD34, CD45, CD29 and CD90) of BMSCs were detected via immunocytochemical staining. The image was observed using a fluorescence microscope (Olympus, Japan).

### Isolation and purification of exosomes from BMSCs

After the cells were cultured in 10% FBS (EV-free) medium for 48 h, we harvested the culture supernatant. Afterwards, the exosomes were extracted using a total exosome isolation reagent kit (Invitrogen) in accordance with the instructions of the manufacture. Subsequently, we resuspended the exosomes in PBS.

### Identification and quantification of exosomes

The morphology of exosomes was observed under a transmission electron microscopy (TEM; Hitachi H-7650, Japan). Later on, the size distribution of exosomes was identified using Nanoparticle tracking analysis (NTA; Nano-Sight LM10; Germany) with particular parameters that were set based on specifications of the manufacturer. In brief, we carried out the exosomal quantification using method described earlier [21]. Through the above-mentioned NTA, we captured and analyzed the exosomes with built-in Nano-Sight software. In view of the visibility of all the particles at 14 without signal saturation, we set the camera at this level before fixing the detection threshold at 5 to ensure exclusion of distinct particles and inclusion of most of the observed ones. Later on, we obtained a final volume of 1 mL after dilution with PBS prior to adjusting the concentration by observing a rate of particles/frame of approximately 50 (30–100 particles/frame). Under the following conditions, namely syringe speed (22 µL/s) and temperature (25 °C), we consecutively recorded five 60 s videos for each measurement. Afterwards, we detected the particles (EVs) with a scientific CMSOS camera and laser (488 nm, blue). Subsequently, we obtained measurements like mean size, particles/ml and most represented EVs size population (mode). Afterwards, the exosomal markers CD29 (Abcam, China), CD44 (Abcam, China), CD45 (Abcam, China) and CD73 (Abcam, China) were determined with flow cytometry.

### Synthesis of DSPE-PEG-CMP

In order to protect CMP from degradation, we modified CMP via covalently bonded PEGs with the reaction being performed as reported previously [22]. Through nucleophilic substitution between NHS amino and CMP N-terminal groups, we synthesized DSPE-PEG-CMP copolymer. Briefly, a 1:2 molar ratio of DSPE-PEG-NHS and CMP were dissolved in dry dimethyl-formamide (DMF), and triethylamine, while the pH was adjusted to 8.2. Later, the reaction proceeded with stirring for 24 h at room temperature. Subsequently, the conjugation efficiency was measured using high-performance liquid chromatography (HPLC) at 220 nm wavelength. The mobile phase consisted of Buffer A ((deionized water with 0.1% trifluoroacetic acid (TFA)) and Buffer B (acetonitrile with 0.1% TFA) at a flow rate of 0.6 mL/min. A linearly gradient (from 0 to 50%) eluate B was used for additional 30 min. After the reaction time, the mixture was dialyzed for 48 h against ultrapure water in a dialysis bag (MWCO 3000 Da). Ultimately, the product was lyophilized and stored at − 20 °C. The DSPE-PEG-NHS and CMP conjugates were identified using the MALDI-TOF-MS mass spectrometer.

### Construction of DSPE-PEG-CMP-EXO

This experiment was performed as described earlier [23]. In brief, DSPE-PEG-CMP was dissolved in anhydrous ethanol at a 5 μM stock concentration. Afterwards, 4 μL of 100 nM ethanolic solution of phospholipid were incubated with the exosomes (200 μL, 1 × 10^10^ particles) for 10 min through gentle rotation at 4 °C. The excess phospholipid was removed via ultrafiltration (MWCO 100 kDa).

### Exosome uptake assay

To investigate whether DSPE-PEG-CMP-EXO could target cardiomyocyte more efficiently, the DSPE-PEG-CMP-EXO and control-exosomes were labeled with DiI and co-cultured with the H9C2 cells for 12 h. Afterwards, the cells were fixed in 4% paraformaldehyde at room temperature for 20 min. Lastly, the nucleus was stained with DAPI. The uptake of exosomes by H9C2 cells was observed under a confocal laser-scanning microscopy (Leica, Japan).

### MiRNA loading via electroporation

To explore the optimal loading ratio of miRNA and engineered exosomes, different concentrations (2.5 to 40 μM) of miR302 mimic was mixed with 1 mg of total engineered exosomes in electroporation buffer. After electroporation (at 350 V, 150 μF) using a Gene Pulser system (Bio-Rad) in a 4 mm cuvette, the mixture was incubated for 30 min at 37 °C to allow the recovery of the exosome membrane. Then, the unloaded miR302 was removed with ultracentrifugation technique (120,000×*g*, 70 min) at 4 °C. Subsequently, the exosome was resuspended in PBS. The loading ratio was evaluated with agarose gel electrophoresis.

### RNA agarose gel electrophoresis

To investigate the stability of engineered-miRNA-exosomes, the DSPE-PEG-CMP-miR302-EXO and miR302 mimic were incubated in DMEM containing 10% FBS at designated time points (0, 2, 4, 8, 12, 24, 36, 48 and 72 h) at 37 °C. After incubation, the samples were mixed with 1% SDS loading buffer and loaded into a 2% agarose gel in 0.5X TBE. Afterwards, we performed electrophoresis at 90 V for 35 min. The bands were imaged with a BioDoc-It imaging system (UVP, USA).

### Quantifying the release of miR302 from DSPE-PEG-CMP-miR302-EXO

To quantify the release of miR302 from DSPE-PEG-CMP-miR302-EXO, 1 mL of DSPE-PEG-CMP-miR302-EXO (20 μg/mL) was dissolved with 50 ml PBS in a dialysis bag (MWCO 3000 Da). Later on, the reaction was proceeded at the shaking speed of 100 rpm at 37 °C. Subsequently, a total of 200 μL mixture was collected at designated time points (1,2, 3, 4, 5, 6, 8, 12, 24, 36 and 48 h) and stored at 4 °C. Finally, the mixtures were analyzed using RiboGreen assay to quantify the amount of miR302 released in solution.

We calculated the percentage cumulative release at each time point by dividing the miR302 released by the miR302 loaded initially.

### Quantitative reverse transcription polymerase chain reaction (RT-PCR)

Total RNA was extracted from H9C2 cells, serum and heart samples using TRIzol reagent (Invitrogen), before it was reverse transcribed into cDNA using a miScript II RT Kit (QIAGEN) as directed by the manufacturer. The level of miR302 was measured with miScript SYBR Green Kit (QIAGEN). The primer sequences can be found in Table 1. MiR302 expression was normalized using U6.

**Table 1:** Primer sequences for quantitative polymerase chain reaction (qRT-PCR)

Target**s**	Sequences (5ʹ-3ʹ)
miR302	F: TCGCTTAAGTGCTTCCATGTTT
	R: CAGTGCGTGTCGTGGAGT
U6	F: GCTTCGGCAGCACATATACTAAAAT
	R: CGCTTCACGAATTTGCGTGTCAT

### ELISA assay

The serum samples were used to determine the level of cardiac troponin I (cTnI), creatine kinase MB (CKMB), tumor necrosis factor-alpha (TNF-α), and interleukin (IL)-1 beta (β) using ELISA kits (Solaibao). The assay was conducted according to manufacturer's instructions.

### Establishment of myocardial I/R model

H9c2 cardiomyocytes were obtained from the Cell Bank of the Chinese Academy of Sciences (Shanghai, China). The H9c2 cells were accordingly pretreated with DSPE-PEG-CMP, DSPE-PEG-CMP-EXO, DSPE-PEG-CMP-miR302-EXO and miR302. When H9c2 cardiomyocytes reached confluency, the normal medium was replaced with serum-free and sugar-free DMEM. As described elsewhere [24], the cells were then incubated at 37 °C in an anaerobic glove box (5% CO_2_ and 95% N_2_) for 6 h. After hypoxia, the medium was replaced with fresh medium before the cells were transferred to the regular incubator for 16 h to mimic reperfusion. Control cells were incubated under normoxic conditions.

### MTT assays

Cell proliferation was measured using MTT assays. The cells were plated in a 96-well plate with density of 1 × 10^4^ cells per well. After reperfusion, 20 µL of MTT (5 mg/mL) was added to the wells and incubated for 4 h. Afterwards, the medium was removed, and 150 μL of DMSO was added to each well. Next, the plates were shaken for 15 min, and the absorbance was recorded at 570 nm by a microplate reader (Bio-Rad).

### Mouse myocardial I/R model and exosome injection

Thirty male C57BL/6 mice (8–10 weeks; 20–25 g) were obtained from Yangzhou University. They were randomly divided into six groups, namely control, model, A (DSPE-PEG-CMP), B (DSPE-PEG-CMP-EXO), C (DSPE-PEG-P-miR302-EXO, and D groups (miR302). The mice were anesthetized using inhalation of 1.5–2% isoflurane and underwent tracheal intubation. After anesthesia, the hearts were exposed and the left anterior descending (LAD) coronary artery was ligated with an 7–0 silk ligature. After occlusion for 60 min, the coronary artery was reperfused by removing the knot of suture. Mice in the control group received the same procedure except the LAD ligation. The animal procedures were approved by the Animal Ethics Committee of Yangzhou University.

Engineered exosomes were intravenously injected via the tail vein (0.25 μg/100 μL PBS/mouse) into the mice in the B and C groups after coronary artery ligation, every 2 days for 4 weeks. Meanwhile, mice in A and D groups were treated with 0.25 μg DSPE-PEG-CMP or miR302 mimic in 100 μL PBS, respectively. The experimental treatments were administered at 12 h after reperfusion.

### Cardiac function

To assess cardiac function at day 28 following MI, transthoracic echocardiography was performed. The mice were anesthetized by nose cone with 5% isoflurane but maintained with 2% of the same anesthetic solution. A Vevo2100 system with a 40‐MHz transducer was used to perform transthoracic echocardiography. The transducer was placed in left parasternal and short-axis views where left ventricular (LV) were recorded. Later, we calculated the parameters of LV, which included ejection fraction (EF), fractional shortening (FS), LV Mass Index, LV volume in diastole (LV VOLd), LV volume in systole (LV VOLs), LV anterior wall thickness at the ends of diastole (LVAWd) and systole (LVAWs), LV end-diastolic internal diameter (LVIDd), LV end-systolic internal diameter (LVIDs), LV posterior wall thickness at the ends of diastole (LVPWd) and systole (LVPWs).

### Histology staining

All the mice were euthanized at day 28 after MI. Then, the heart samples were fixed in 4% paraformaldehyde for 24 h, dehydrated, embedded in paraffin, and sectioned at 5 μm thickness. Subsequently, the sections were stained with hematoxylin–eosin (HE). The histopathological changes were observed using a light microscope (Olympus, Japan).

### TUNEL staining

The cellular death was evaluated with terminal deoxynucleotidyl transferase-mediated dUTP nick-end labeling (TUNEL) staining. The TUNEL assay was performed using a commercial kit (Roche, China) according to the manufacturer’s instructions. Staining was observed under a fluorescence microscopy, while TUNEL-positive nuclei were calculated as a percentage (%) of total nuclei.

### Western blotting

Cells and heart tissues were lysed in lysis buffer containing protease inhibitors. Later on, the proteins were separated via 10% SDS-PAGE and transferred to a PVDF membrane. Next, the membranes were blocked in 5% milk for 2 h, before their incubation with the anti-Yap (Affinity Biosciences, cat. no. DF3182; 1:1000; China), anti-Ki67 (Affinity Biosciences, cat. no. AF0198; 1:2000; China), anti-Bax (Abclonal, cat. no. A19684; 1:2000; China), anti-Bcl2 (ABclonal, cat. no. A19693; 1:1000; China), and anti-GAPDH (Affinity Biosciences, cat. no. AF7021; 1:3000; China) at 4 °C overnight. The membranes were washed with TBST and incubated with HRP-conjugated anti-rabbit IgG (Affinity Biosciences, cat.no. S0001; 1:3000; China) for 2 h. The proteins were visualized by enhanced chemical luminescence reagents. The band intensity was analyzed with the software of Image J.

### Statistical analysis

All data were processed using GraphPad Prism7.0. The experiments were all replicated at least three times. Unpaired two-tailed Students t-test was used for comparison between two groups. For comparison between multiple groups, one-way ANOVA was performed. Data were exhibited as mean ± standard deviation. *P* < 0.05 was recognized as significant.

## Results

### Isolation and identification of BMSCs

**Fig. 1:**
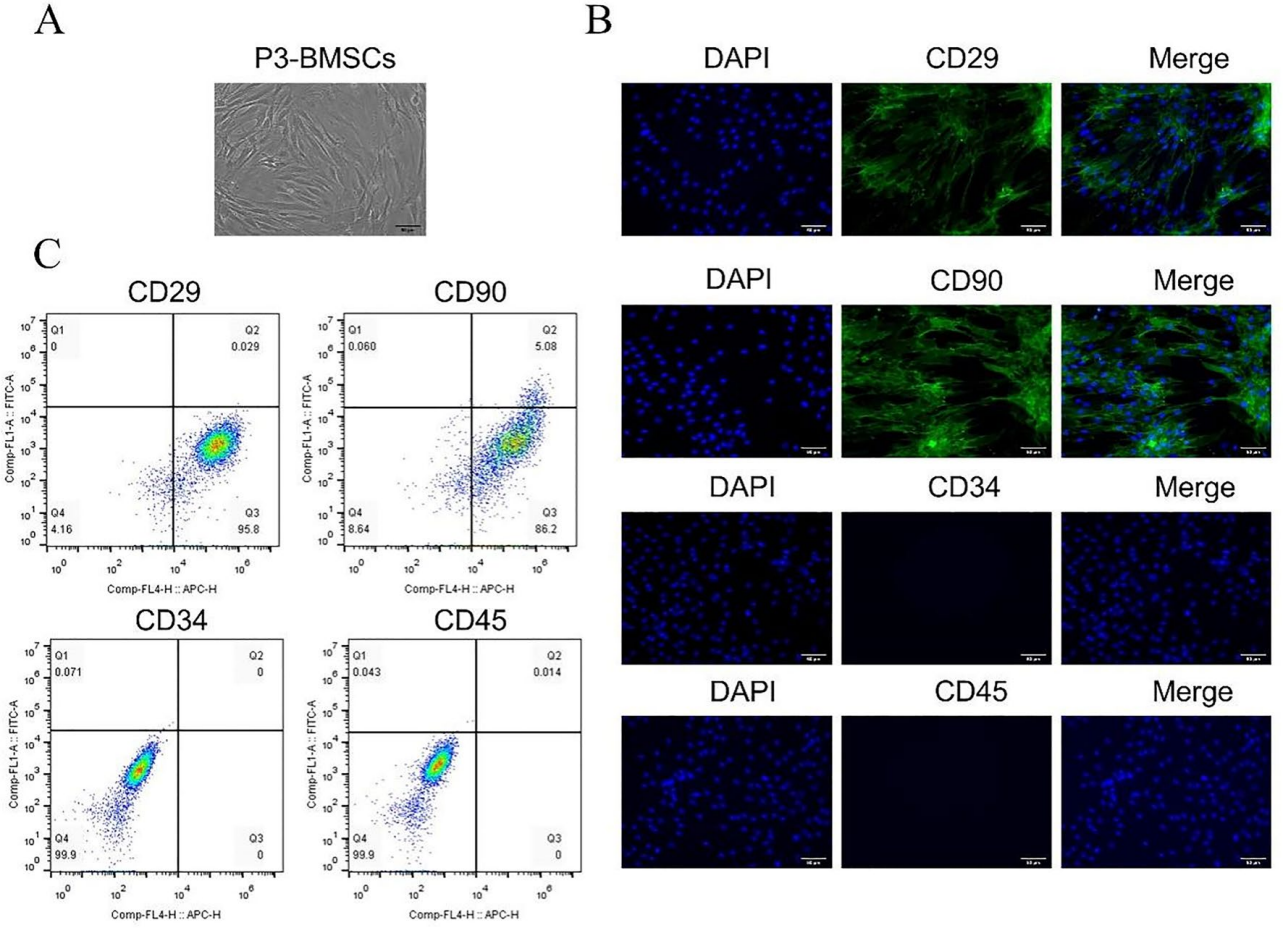
Isolation and identification of bone marrow mesenchymal stromal cells (BMSCs). **A** The morphological characteristics of BMSCs observed under a inverted phase contrast microscopy. Scale bar = 50 μm. **B** Immunofluorescent technique was employed to analyze the surface markers (CD34, CD45, CD29 and CD90) of BMSCs. Scale bar = 50 μm. **C** Flow cytometric technique was applied to analyze surface markers (CD34, CD45, CD29 and CD90) of BMSCs

The morphological features of BMSCs were observed under inverted phase contrast microscopy. After passaging to the third generation, majority of the BMSCs displayed long spindle-like morphology and whirl-like growth (Fig. 1A). Moreover, immunofluorescence results showed the expression of mesenchymal markers CD29(+), CD90(+), CD34(−) and CD45(−) (Fig. 1B). Flow cytometry also indicated that these cells were positive for stromal cell markers CD29 (95.8%) and CD90 (86.2%), however, almost no expression of the hematopoietic markers like CD34 and CD45 (Fig. 1C). These findings suggest that the cells tested were purified BMSCs.

### Isolation and characterization of BMSCs-EXO, DSPE-PEG-CMP-EXO and DSPE-PEG-CMP-miR302-EXO

**Fig. 2:**
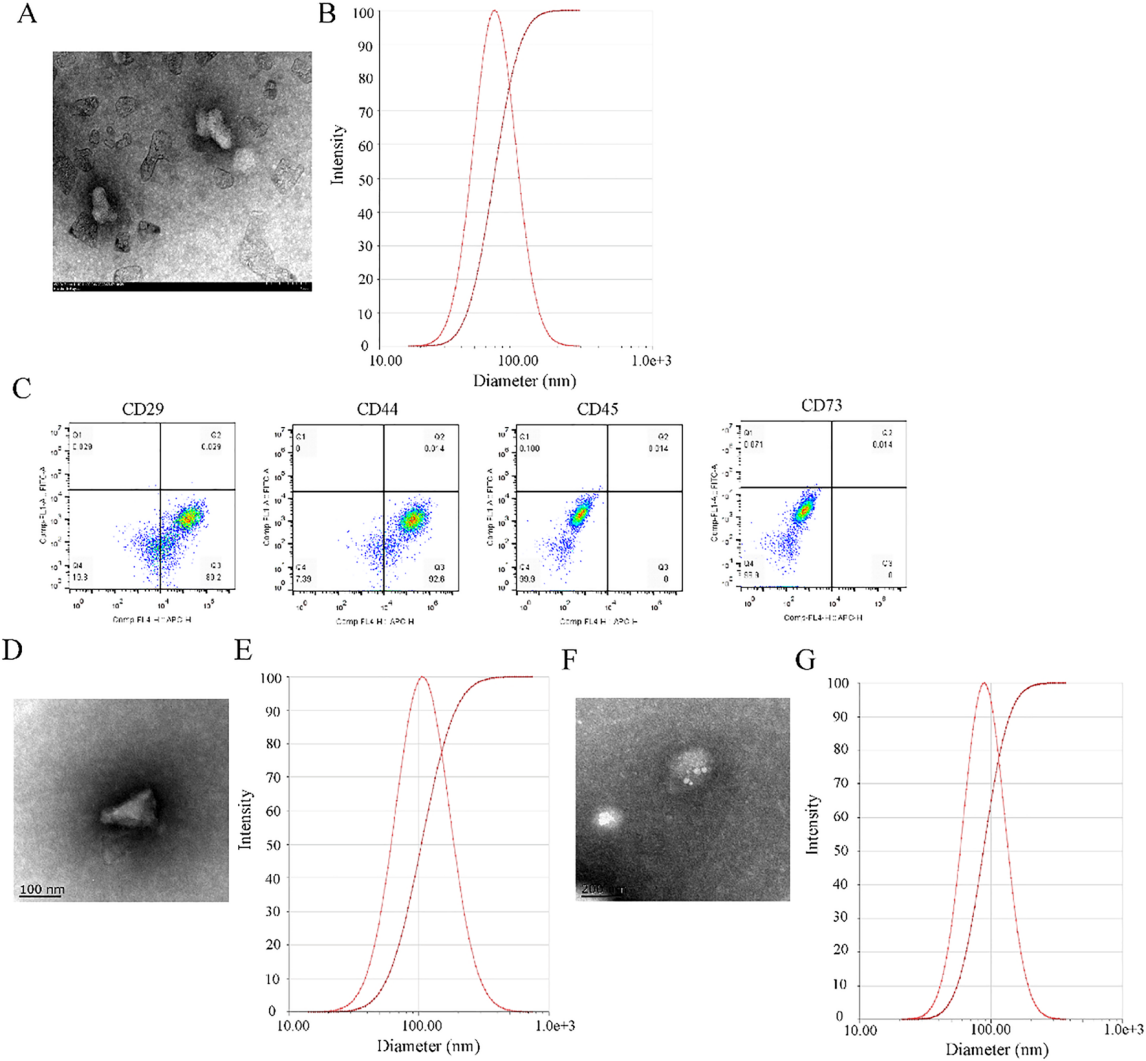
Isolation and identification of exosomes derived from bone marrow mesenchymal stromal cells (BMSCs, BMSCs-EXO), as well as synthesis and characterization of DSPE-PEG-CMP-EXO and DSPE-PEG-CMP-miR302-EXO. **A** Morphological characteristics of BMSCs-derived exosomes (BMSCs-EXO) was observed under transmission electron microscope (TEM). Scale bar = 100 nm. **B** The size distribution of BMSCs-EXO was measured via the Nanoparticle tracking analysis (NTA). **C** Flow cytometric technique was used to analyze the specific surface markers (CD44, CD45, CD29 and CD73) of BMSCs of BMSCs-EXO. Scale bar = 100 nm. **D** TEM morphology of DSPE-PEG-CMP-EXO. **E** NTA measured size distribution of DSPE-PEG-CMP-EXO. **F** TEM morphology of DSPE-PEG-CMP-miR302-EXO. Scale bar = 200 nm. **G** The size distribution of DSPE-PEG-CMP-miR302-EXO was measured with the NTA

Exosomes were isolated from the culture medium of BMSCs. The TEM image showed that the morphology of BMSCs-EXO was cup- or sphere-shaped (Fig. 2A), while the NTA results showed the average particle size of BMSCs-EXO to be 69.31 ± 0.44 nm (Fig. 2B). Moreover, flow cytometry also indicated that these exosomes were positive for stromal cell markers CD29 (80.2%) and CD44 (92.6%), but almost no expression of the hematopoietic marker CD45 and CD73 was observed (Fig. 2C). These results suggest successful isolation of EXO from BMSCs successfully. In addition, the TEM image showed that the morphology of DSPE-PEG-CMP-EXO was saccular-shaped (Fig. 2D), and the NTA results showed the average particle size of DSPE-PEG-CMP-EXO to be 105.16 ± 1.44 nm (Fig. 2E); As shown in Fig. 2F, G the morphology of DSPE-PEG-CMP-miR302-EXO was sphere-shaped, and the average particle size of DSPE-PEG-CMP-miR302-EXO to be 137.36 ± 2.84 nm.

### Synthesis and identification of DSPE-PEG-CMP

**Fig. 3:**
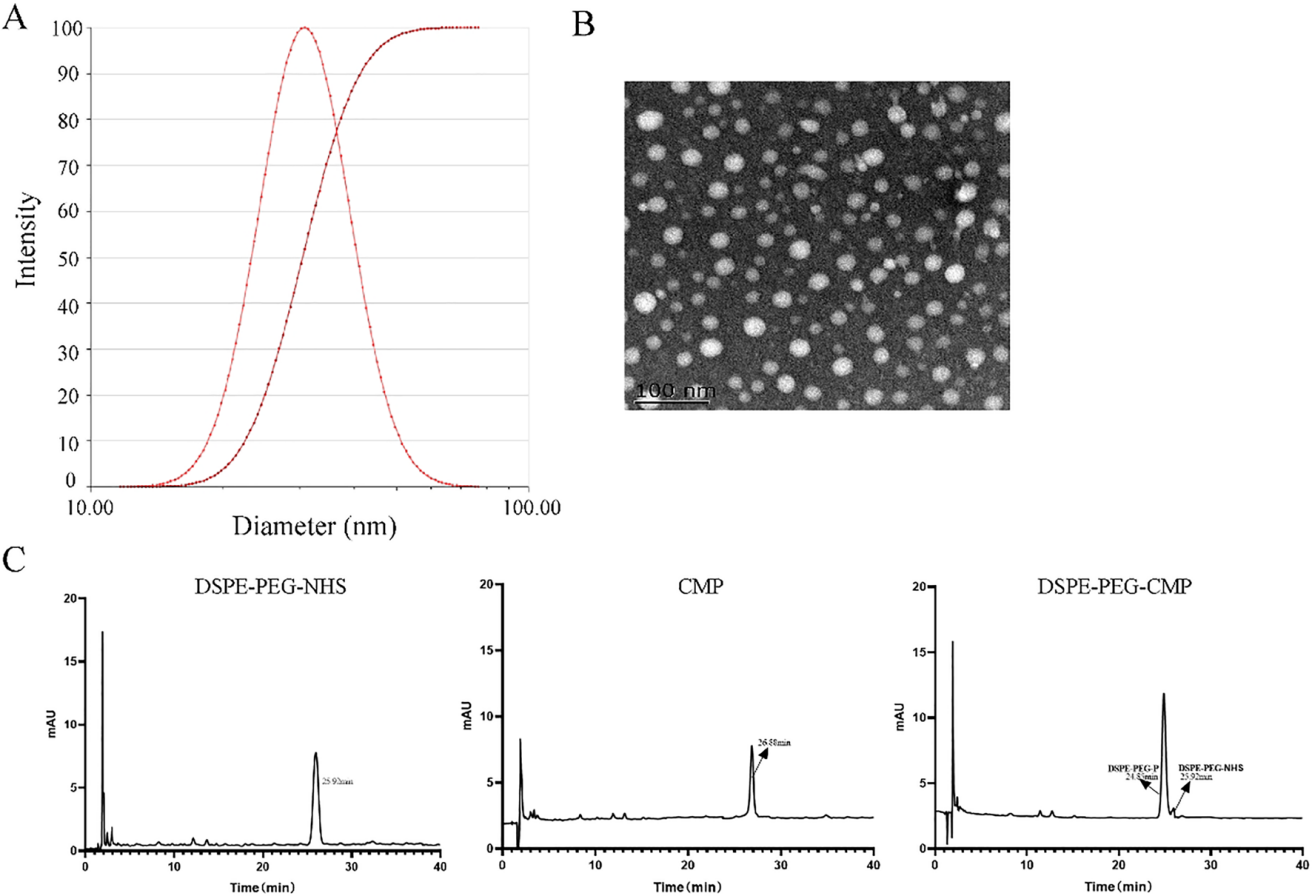
Characterization and analysis of DSPE-PEG-CMP. **A** The size distribution of DSPE-PEG-CMP was measured with the Nanoparticle tracking analysis (NTA). **B** TEM morphology of DSPE-PEG-CMP. Scale bar = 100 nm. **C** The high-performance liquid chromatography (HPLC) chromatogram of DSPE-PEG-NHS, CMP and DSPE-PEG-CMP. mAu: milli Absorbance unit

The DSPE-PEG-CMP copolymer was synthesized through nucleophilic substitution between NHS amino group and CMP N-terminal group. The NTA results showed average particle size of DSPE-PEG-CMP was 30.08 ± 0.62 nm (Fig. 3A). As shown via TEM technique, the morphology of DSPE-PEG-CMP was spheroid-like with the particle sizes around 30 nm (Fig. 3B), which is in line with NTA results. According to the HPLC chromatogram, the unreacted DSPE-PEG- NHS and CMP retention times were respectively 25.92 min and 26.88 min, which displayed good peak shape with no interfering peaks. Moreover, the unreacted DSPE-PEG-CMP retention time was 24.85 min, amid demonstration of larger molecular weight (Fig. 3C). These results indicate that the DSPE-PEG-NHS coupled with CMP successfully.

### Measurement of quantity and stability of miR302 loading into DSPE-PEG-CMP-EXO

**Fig. 4:**
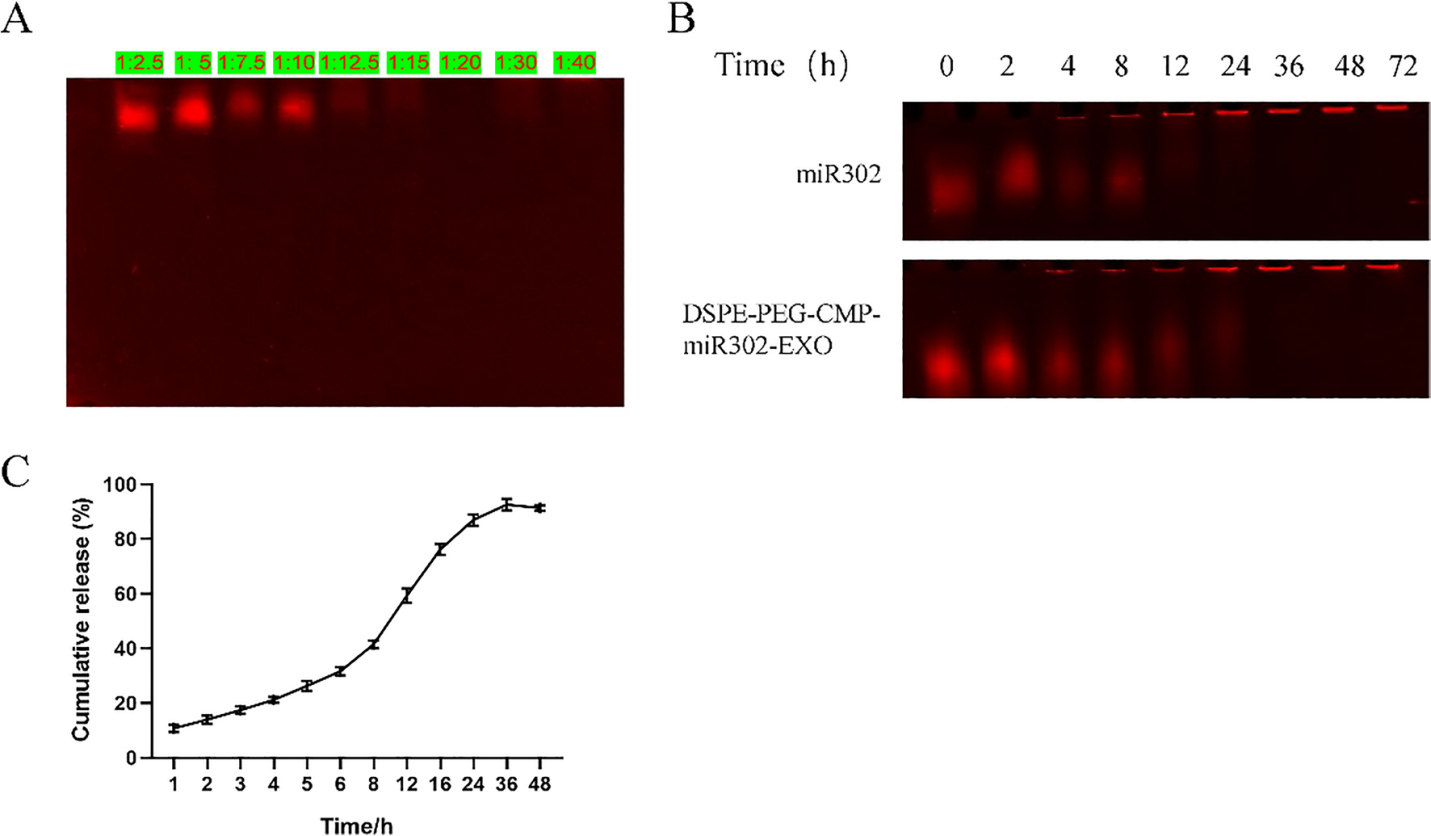
Measurement of quantity and stability of miR302 that was loaded into DSPE-PEG-CMP-EXO. **A** Agarose gel electrophoresis showed that miR302 was efficiently loaded into DSPE-PEG-CMP-EXO at 20 µM. **B** The stability of DSPE-PEG-CMP-miR302-EXO was evaluated using agarose gel electrophoresis, wherein miR302 was stably expressed for 24 h. **C** The cumulative release was measured with RiboGreen assay, which showed sustained release

In order to investigate the most efficient electroporation-mediated loaded miRNA in DSPE-PEG-CMP-EXO, we used different concentrations of miR302 mimic (2.5 to 40 μM), and electroporated them with the same amount of DSPE-PEG-CMP-EXO (1 mg). The amount of miR302 mimic loaded into DSPE-PEG-CMP-EXO was evaluated via agarose gel electrophoresis. We found that the miR302 mimic concentrations of 20 μM resulted in efficient encapsulation of DSPE-PEG-CMP-EXO (Fig. 4A). To evaluate stability of DSPE-PEG-CMP-miR302-EXO, the same concentration of miR302 mimic was incubated in DMEM with 10% FBS at different time points (0 to 72 h). Also, the stability of DSPE-PEG-CMP-miR302-EXO was evaluated with agarose gel electrophoresis. It was observed that the miR302 mimic was stably expressed only for 12 h, while miR302 mimic loaded to DSPE-PEG-CMP-EXO was stably expressed for 24 h (Fig. 4B). Later on, we quantified the release of miR302 from DSPE-PEG-CMP-miR302-EXO. As detected via RiboGreen assay, at the end of 48 h, 90% cumulative release was observed (Fig. 4C). These results indicate that DSPE-PEG-CMP-EXO complex has strong chelating, releasing ability and good stability for miR302.

### In vitro function of DSPE-PEG-CMP-miR302-EXO

**Fig. 5:**
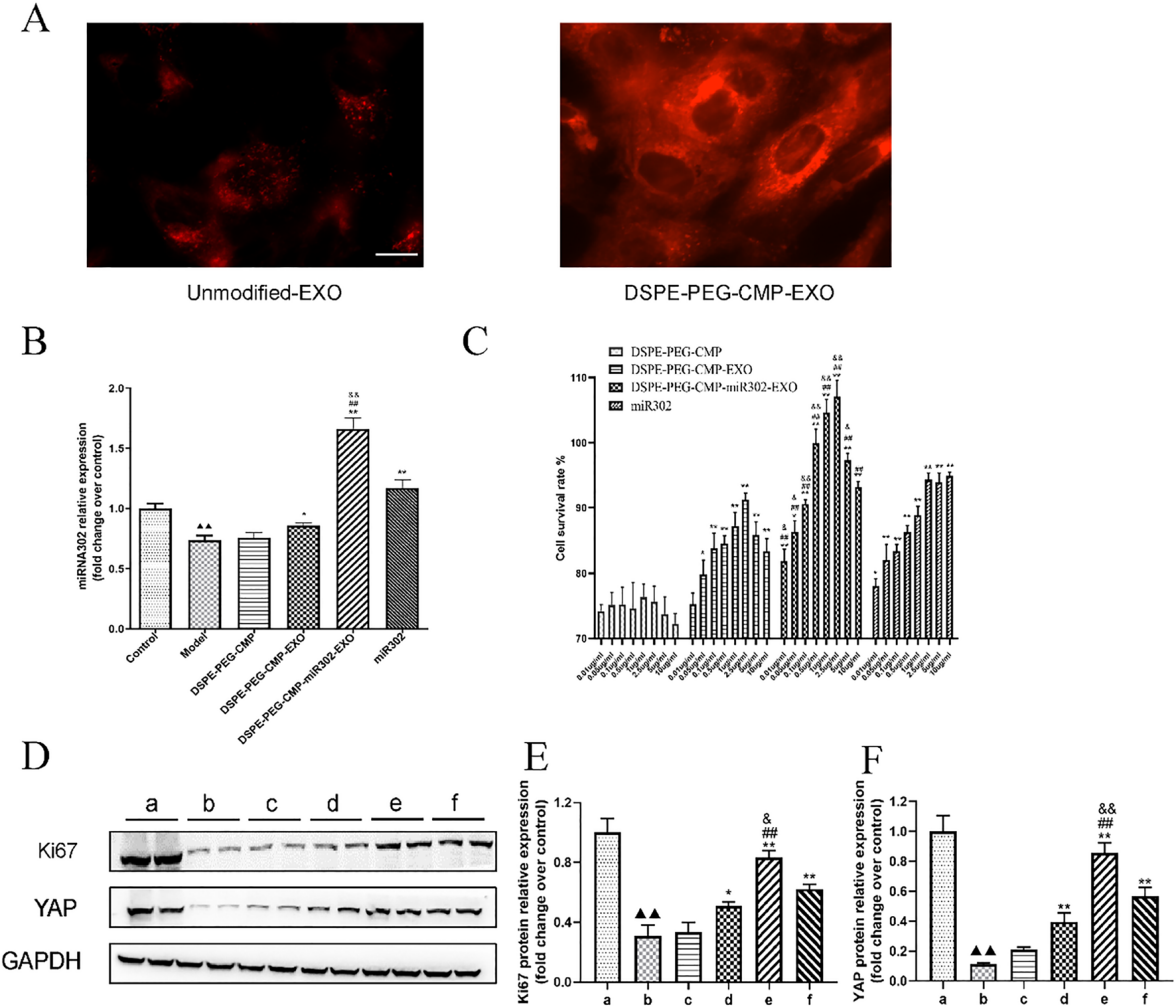
The in vitro uptake and effect of DSPE-PEG-CMP-miR302-EXO on gene expression in H9C2 cells. **A** Fluorescence images indicated internalization of BMSCs-EVs and DSPE-PEG-CMP-miR302-EXO in H9C2 cells. Scale bar = 50 μm. **B** Quantitative polymerase chain reaction (qRT-PCR) indicated upregulation of miR302 level in H9C2 cells by DSPE-PEG-CMP-miR302-EXO. Relative values were presented as fold change over control. **C** The MTT assay showed increased proliferation ratio of H9C2 cells by DSPE-PEG-CMP-miR302-EXO. **D**–**F** Western blot analysis of Ki67 and Yap levels displayed increased levels of these proteins in cardiomyocytes after co-incubation with DSPE-PEG-CMP-miR302-EXO. GAPDH served as the internal reference. Relative values were presented as fold change over control. a: Control; b: Model; c: DSPE-PEG-CMP; d: DSPE-PEG-CMP-EXO; e: DSPE-PEG-CMP-miR302-EXO; f: miR302. **B**, **E**, **F** ^▲▲^p < 0.01 vs control; *p < 0.05, **p < 0.01 vs Model; ^##^p < 0.01 vs DSPE-PEG-CMP-EXO; ^&^p < 0.05, ^&&^p < 0.01 vs miR302. **C** *p < 0.05, **p < 0.01 vs DSPE-PEG-CMP; ^##^p < 0.01 vs DSPE-PEG-CMP-EXO; ^&^p < 0.05, ^&&^p < 0.01 vs miR302

To investigate whether DSPE-PEG-CMP-EXO was more readily endocytosed by cardiomyocytes than unmodified-EXO, H9c2 cardiomyocytes were incubated with DiI labeled unmodified-EXO or DSPE-PEG-CMP-EXO and imaged at 12 h. The result showed that cardiomyocytes exhibited increased uptake of DSPE-PEG-CMP-EXO when compared with unmodified-EXO (Fig. 5A). Next, we investigated the effects of DSPE-PEG-CMP-miR302-EXO on myocardial I/R injury. According to qRT-PCR result, the level of miR302 in model group was significantly decreased compared to control group (*p* < 0.01). Moreover, the miR302 level was marked higher in DSPE-PEG-CMP-miR302-EXO treated cells compared to miR302 mimic treated cells (*p* < 0.01) (Fig. 5B). Furthermore, we explored the proliferation-promoting function of DSPE-PEG-CMP-miR302-EXO. The MTT result showed a reduction in the rate of cell proliferation at 5 to 10 μg/mL of DSPE-PEG-CMP, thus indicating that high concentration of DSPE-PEG-CMP exhibited some cytotoxicity. In addition, the cell proliferation rate showed a statistically increase in DSPE-PEG-CMP-miR302-EXO treated cells compared to PEG-CMP-EXO and miR302 mimic treated cells (*p* < 0.01) (Fig. 5C). As shown via western blot technique, DSPE-PEG-CMP-miR302-EXO significantly increased the Ki67 and Yap protein levels compared to DSPE-PEG-CMP-EXO and miR302 mimic group (*p* < 0.01) (Fig. 5D–F). These results showed that DSPE-PEG-CMP-EXO loaded with miR302 can significantly promote H9c2 cardiomyocytes proliferation.

### DSPE-PEG-CMP-miR302-EXO treatment significantly inhibited inflammatory response and IR injury

**Fig. 6:**
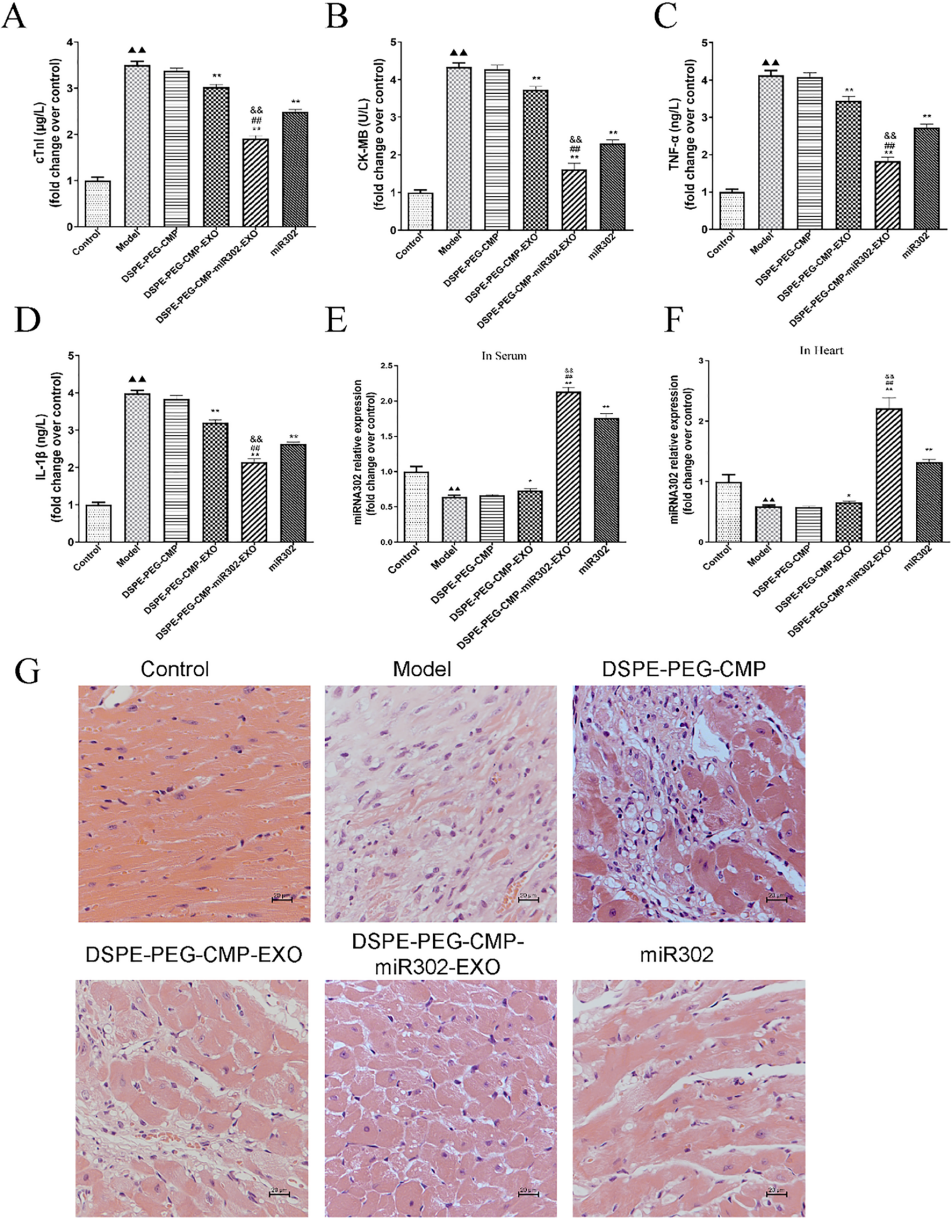
DSPE-PEG-CMP-miR302-EXO treatment significantly inhibited inflammatory response and IR injury. **A**–**D** ELISA showed that DSPE-PEG-CMP-miR302-EXO decreased levels of cTnI, CKMB, TNF-α and IL-1β in cardiomyocytes. **A** cTnI: cardiac troponin I; **B** CKMB: creatine kinase MB; **C** TNF-α: tumor necrosis factor-alpha; **D** IL-1β: interleukin (IL)-1 beta (β). **E**, **F** The miR302 level in serum and heart was detected with qRT-PCR, wherein the level was higher in DSPE-PEG-CMP-miR302-EXO group. Relative values were presented as fold change over control. **G** HE staining image showed reduced heart necrosis in the myocardial I/R injury after DSPE-PEG-CMP-miR302-EXO treatment. Scale bar = 100 μm

To evaluate the treatment effect of DSPE-PEG-CMP-miR302-EXO in vivo, a cardiomyocyte H/R mouse model was established. Later, the mice were treated with DSPE-PEG-CMP-miR302-EXO every 2 days for 4 weeks via tail veins. Levels of the myocardial injury markers (cTnI and CKMB) and proinflammatory factors (TNF-α and IL-1β) in serum were detected by ELISA. As expected, DSPE-PEG-CMP-miR302-EXO significantly decreased the levels of cTnI, CKMB, TNF-α, and IL-1β compared to DSPE-PEG-CMP-EXO and miR302 mimic group (*p* < 0.01) (Fig. 6A–D). Moreover, the levels of miR302 in serum and heart were detected in all groups by qRT-PCR technique. We discovered that level of miR302 in DSPE-PEG-CMP-miR302-EXO group was higher compared to DSPE-PEG-CMP-EXO and miR302 mimic group (*p* < 0.01) (Fig. 6E, F). HE staining showed that cardiomyocytes were disarrayed with severe necrosis and intense inflammatory infiltrate, and cardiac structural integrity was compromised in IR-treated mice. However, DSPE-PEG-CMP-miR302-EXO treatment remarkably reduced the degree of cardiomyocytes necrosis and alleviated cardiomyocyte disorder (Fig. 6G).

**Fig. 7:**
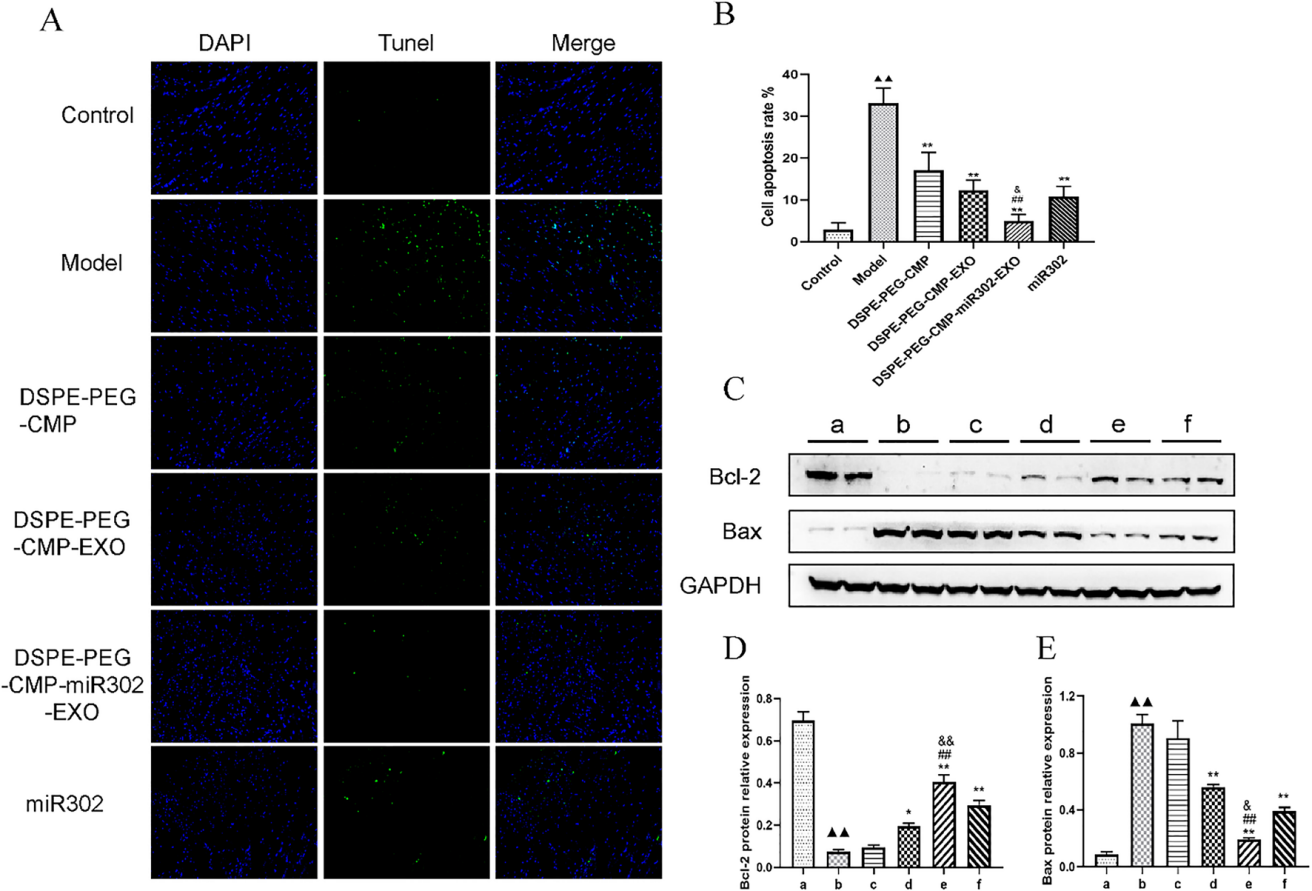
DSPE-PEG-CMP-miR302-EXO treatment significantly reduced myocardial apoptosis. **A** TUNEL staining (Nuclei was stained blue with DAPI) demonstrated that cardiomyocytes underwent apoptosis. Scale bar = 100 μm. **B** DSPE-PEG-CMP-miR302-EXO reduced the apoptotic rate of cardiomyocyte (TUNEL-positive cells (%) are expressed as number of apoptotic myocytes/total myocytes). **C**–**E** Western blot analysis showed that DSPE-PEG-CMP-miR302-EXO upregulated Bcl-2 expression but inhibited that of Bax in cardiomyocytes. GAPDH served as the internal reference. Values are expressed as mean ± SD (n = 3). a: Control; b: Model; c: DSPE-PEG-CMP; d: DSPE-PEG-CMP-EXO; e: DSPE-PEG-CMP-miR302-EXO; f: miR302. ^▲▲^p < 0.01 vs control; *p < 0.05, **p < 0.01 vs Model; ^##^p < 0.01 vs DSPE-PEG-CMP-EXO; ^&&^p < 0.01 vs miR302

Furthermore, DSPE-PEG-CMP-miR302-EXO treatment significantly reduced the myocardial apoptosis rates compared to DSPE-PEG-CMP-EXO and miR302 mimic group (*p* < 0.01), which was confirmed by the TUNEL staining (Fig. 7A, B). In addition, Western blot analysis showed that DSPE-PEG-CMP-miR302-EXO significantly upregulated Bcl-2 expression but decreased that of Bax compared to DSPE-PEG-CMP-EXO and miR302 mimic group (p < 0.01) (Fig. 7C–E).

### DSPE-PEG-CMP-miR302-EXO treatment significantly preserved cardiac function

**Fig. 8:**
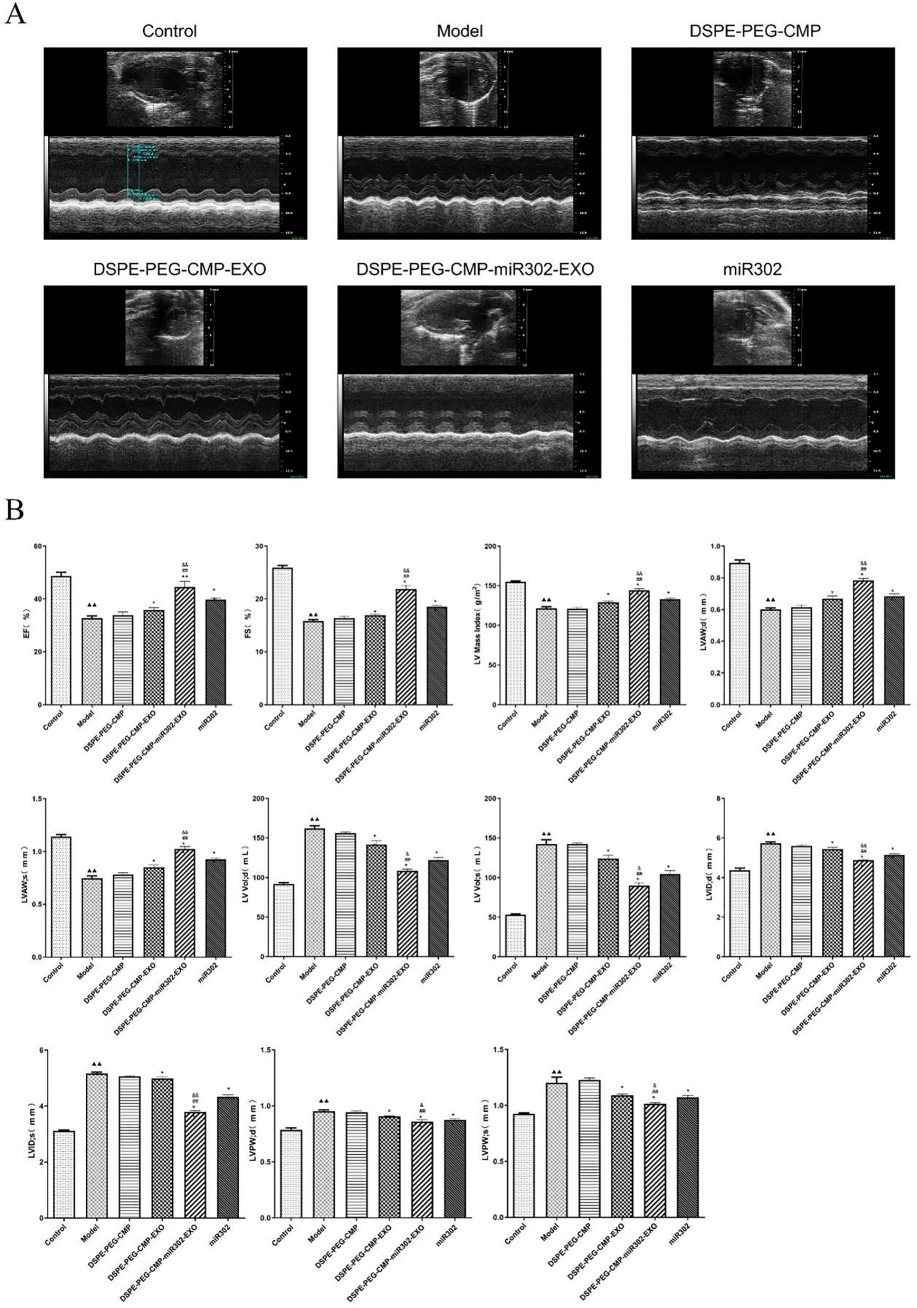
DSPE-PEG-CMP-miR302-EXO treatment significantly preserved cardiac function. **A** Echocardiographic images of mice with myocardial I/R injury after DSPE-PEG-CMP-miR302-EXO treatment showed improved cardiac function. **B** Increased EF, FS, LV Mass Index, LV VolD, LV VolS, and decreased LVAWd, LVAWs, LVIDd, LVIDs, LVPWd and LVPWs of mice with myocardial I/R injury were observed after DSPE-PEG-CMP-miR302-EXO treatment. *EF* ejection fraction, *FS* fractional shortening, *LV Mass Index* left ventricular mass index, *LV VOLd* LV volume in diastole, *LV VOLs* LV volume in systole, *LVAWd* LV anterior wall thickness at the ends of diastole, *LVAWs* LV anterior wall thickness at the ends of systole, *LVIDd* LV end-diastolic internal diameter, *LVIDs* LV end-systolic internal diameter, *LVPWd* LV posterior wall thickness at the ends of diastole, *LVPWs* LV posterior wall thickness at the ends of systole. ^▲▲^p < 0.01 vs control; *p < 0.05, **p < 0.01 vs Model; ^##^p < 0.01 vs DSPE-PEG-CMP-EXO; ^&&^p < 0.01 vs miR302

Additionally, we detected the effect of DSPE-PEG-CMPmiR302-EXO on cardiac function. Cardiac function was examined using echocardiography on postoperative day 28 (Fig. 8A). Echocardiography results revealed that EF, FS, LV Mass Index, LVAWd and LVAWs were significantly decreased, whereas LV VOLd, LV VOLs, LVIDd, LVIDs, LVPWd and LVPWs were noticeably increased in the model group compared with control group (p < 0.01). Meanwhile, DSPE-PEG-CMP-miR302-EXO significantly increased the EF, FS, LV Mass Index, LVAWd and LVAWs and decreased the LV VOLd, LV VOLs, LVIDd, LVIDs, LVPWd and LVPWs compared to model group (p < 0.01) (Fig. 8B).

## Discussion

Recently, scientists have identified MSC as one of the most powerful therapeutic approaches to repair myocardial injury [25]. In the present study, we developed an engineered BMSCs-EXOs, which was modified with a CMP and loaded with miR302 to treat myocardial I/R injury in vitro and in vivo. We found that DSPE-PEG-CMP-miR302-EXO remarkably promoted H9C2 cells proliferation in vitro*.* Also, DSPE-PEG-CMP-miR302-EXO amplified and enhanced the anti-inflammatory, anti-apoptosis and protective effects on cardiac function in vivo. Our data showed that DSPE-PEG-CMP-miR302-EXO has great potential for H9C2 internalization and treatment of myocardial I/R. It is possible that therapeutic potential of DSPE-PEG-CMP-miR302-EXO may partly be attributed to miR302 since existing literature has indicated that overexpression of the microRNA could substantially promote cardiomyocyte apoptosis [26]. In another work, Wu and colleagues affirmed that cardiomyocyte proliferation was promoted by miR302 via regulation of Hippo pathway [27]. Besides, Wang and co-experimenters observed promotion of cardiomyocyte proliferation and regeneration of cardiac function by miR302 [8].

Exosomes are natural nano-sized vesicle, which have several advantages, such as low cytotoxicity, high circulatory stability, and low immunogenicity [28, 29]. Moreover, they can protect transmit substances (such as nucleic acids, drugs) from degradation and enhance intracellular delivery. In addition, exosomes are believed to be promising therapeutic tools for heart damage [30, 31]. For example, MSC-derived exosomes have been demonstrated to reduce myocardial apoptotic, I/R injury and improve cardiac function [32–34]. Thus, ESC-derived exosomes can promote the survival and proliferation of cardiomyocytes for cardiac regeneration [35]. Exosomes, however, are primarily absorbed by the mononuclear phagocyte system after systemic injection, resulting in poor targeting in vivo [20]. To deliver therapeutic exosomes to the target cells or tissues, specific targeting strategies (such as genomic editing of exosome-derived cells, direct modification) can be employed [36, 37]. In this work, we isolated BMSC and its exosomes and identified them using appropriate surface markers. In terms of identification of the BMSCs, we followed the minimum criteria of the Stem Cell Committee of Mesenchymal and Tissue of the International Society for Cellular Therapy, which suggest the use of the classical expressions of negative (CD79α or CD19, CD45, CD34, CD14 or CD11b), positive (CD105, CD90 and CD73) and HLA-DR surface markers [38]. In recent times, various surface markers have been utilized to characterize BMSC, which usually involve a combination of the above-mentioned surface markers. Existing literature has suggested that skin fibroblasts and other plastic adherent type of cells with the ability to propagate umbilical vein endothelial cells in vitro also positively expressed CD73 and CD105, which suggest that the identity of MSC cannot be proven without CD90 [39, 40]. Also, CD73 and CD105 are disadvantaged in terms of a minimal cross-reactivity of anti-human antibodies with animal cells [41]. On the basis of the aforementioned issues with CD73 and CD105, we utilized CD90 to characterize BMSC. Through flow cytometric technique, we observed that the BMSC exosomes positively expressed CD29 and CD44, but almost no expression of the CD45 and CD73. The CD73 is known to be positively expressed on exosomes that have been isolated from various MSCs. However, MSCs (like BMSC) are nonuniform and thus can dynamically change within in vivo local environment and after they have been isolated and cultivated in vitro [42]. It possible that our isolated exosomes displayed low expression of CD73, which may be attributed to factors such as source of BMSC, isolation and cultivation procedure, etc.

Increasingly, peptides are being applied to exosome research in recent years. For example, Pham et al. introduced EGFR-targeting peptides to exosomes surfaces, which improved the targetability of exosomes loaded with antitumor drugs and significantly enhanced their therapeutic effects [43]. Liang et al. attached chondrocyte-affinity peptide to the surfaces of exosomes, which noticeably enhanced exosomes targeting of chondrocytes and alleviated osteoarthritis progression in a rat model. In the present study, we designed a CMP which was displayed on the surface of BMSC-derived exosomes to enhance the specificity and efficiency of delivery to cardiomyocyte. The CMP (WLSEAGPVVTVRALRGTGSW) is a cardiomyocyte‐targeting peptide that was identified through phage display technology, wherein it can specifically recognize tenascin X on cardiomyocytes surfaces and is widely used to modify drugs and their carriers [44–46]. In addition, click chemistry is a promising method of linking peptides directly to the exosome membrane. Available literature suggests that Mentkowski and Lang engineered cardiosphere derived cells (CDCs) exosomes to express CMP and targeted delivery to cardiomyocytes and reduced apoptosis of cardiomyocytes [2]. However, the heterogenous nature of CDCs-exosomes affected its therapeutic effects [47]. On the other hand, clinical studies have established safeness of BMSCs, while their exosomes seem homogenous with the potential to impact several cardiomyocytes pathways which result in decrease of myocardial injury and cardiac function improvement [48]. Also, the multipotent nature of MSCs have made them to potentially exhibit significant regenerative property [49]. Besides, other studies have found that DSPE-PEG can be embedded on the membrane of exosomes. In this regard, we successfully immobilized the DSPE-PEG-CMP on BMSC exosomes. According to the TEM and the NTA results, reconstruction did not affect the physical properties of exosomes. Bounding of DSPE-PEG on exosomes derived from mouse aortic endothelial cells (MAECs) had no influence on physical properties of the aforementioned cells, especially the cell viability [50]. Furthermore, we found that DSPE-PEG-CMP-EXO could be internalized by H9C2 cells more efficiently than control-exosomes and promoted the proliferation of cardiomyocytes remarkably. Nonetheless, increased cardiomyocytes proliferation was not observed, wherein this may be attributed to little capacity for cardiomyocytes of adult mammalian to proliferate in response to injury [51].

It remains a major challenge in delivering miRNA to target cells steadily and effectively, even though miRNA-based therapies have shown promising therapeutic benefits. In our study, we introduced the miR302 to engineered exosomes by electroporation, and the results showed that miR302 was remarkably upregulated in cardiomyocyte, thus suggesting good sustained release and good RNA protection potential of DSPE-PEG-CMP-miR302-EXO. In this work, DSPE-PEG-CMP-miR302-EXO cumulatively released 90% of miR302 at the end of 48 h. As an encapsulation matrix, DSPE-PEG has been found to conjugate with microRNAs via its lipid poly (ethylene glycol) moiety, which improved the RNA segments stability against enzymatic degradation [52]. This phenomenon may have improved sustained release of miR302 for 48 h, albeit further study needed in different media and conditions. Additionally, we found that DSPE-PEG-CMP-miR302-EXO significantly increased the level of Ki67 and Yap, promoted H9C2 cells proliferation in vitro. As a downstream transcriptional effector of Hippo signaling, Yap is translocated to the nucleus to activate proliferation related genes upon loss of Hippo signaling [53]. Previous studies have shown that miR302 inhibited the Hippo signal transduction pathway to promote cardiomyocyte proliferation [9], which is consistent with our findings. In vivo results indicated that DSPE-PEG-CMP-miR302-EXO significantly suppressed the TNF-α, IL-1β and Bax protein levels, thus suggesting anti-inflammatory and anti-apoptosis effect of DSPE-PEG-CMP-miR302-EXO. This finding is consistent with existing literature, which posited that BMSC-exosomes could downregulate caspase-3 and Bax but upregulate BCl-2 as well as reduce levels of inflammatory biomarkers, namely tumor necrosis factor-alpha (TNF-α), interleukin (IL) 1 beta (β) and 6 [54]. The hippo/Yap signaling pathway has been found to crucially mediate apoptosis and oxidative stress, wherein Hippo pathway activation result in Yap inhibition and induction of apoptosis [55, 56]. In this work, DSPE-PEG-CMP-miR302-EXO activated Yap, thus suggesting potential inhibition of apoptosis through suppression of Hippo pathway. However, this phenomenon will be confirmed in our subsequent experiments. Available works have shown that TLR4/MyD88/NF-κB and NLRP3/caspase-1 are principal players in inflammatory response during MI [55]. Although, the mechanism underlying the anti-inflammatory activity of miR302 is not clear, existing literature suggests that the miR302a, a member of miR302 family could suppress inflammation of vascular system through inhibition of nuclear factor-κB (NF- κB) pathway in endothelial cells [57]. Likewise, miR302e (another member of miR302 family) demonstrated anti-inflammatory property by attenuating allergic inflammation via suppression of NF-κB activation [58]. Also, miR302b could regulate activation of NF-κB, which ultimately resulted in alleviation of lung injury [59]. Altogether, these findings suggest that miR302 may suppress inflammation through suppression of NF- κB activation. Notwithstanding, comprehensive research work is needed to confirm this notion. Furthermore, DSPE-PEG-CMP-miR302-EXO further improved cardiac function in a myocardial I/R mice model. These results indicate that the engineered exosomes have excellent transmission of miRNA and cardiac protective functions.

This is a preliminary study of exploring the potential of DSPE-PEG-CMP-miR302-EXO to attenuate myocardial apoptosis and inflammatory response. First, we did not explore the receptor for CMP and the mechanism by which they interact. Moreover, we only confirmed the functional roles of DSPE-PEG-CMP-miR302-EXO, and do not identify the clear mechanism of BMSC-exosomes attenuating myocardial apoptosis and inflammatory response. In addition, we did not explore the effects of miR302 on other pathways during myocardial I/R injury. We will comprehensively investigate the mechanistic details to understand how DSPE-PEG-CMP-miR302-EXO attenuate myocardial apoptosis and inflammatory response in our not-too distant future works. Notwithstanding, some innovative points of this study include (i) incorporation of miR302 into DSPE-PEG-CMP- EXO carrier, which resulted in improved sustained release of the microRNA to H9C2 cells, an vitro model for cardiomyocyte. (ii) Efficient internalization of DSPE-PEG-CMP-EXO by H9C2 cells, which may efficiently and remarkably promote cardiomyocytes proliferation. (iii) Potential increase in protein levels of Ki67 and Yap by DSPE-PEG-CMP-miR302-EXO. (iv) Amplification and enhancement of the anti-inflammatory, anti-apoptosis and cardiac function by DSPE-PEG-CMP-miR302-EXO.

## Conclusion

In summary, we discovered that BMSC-derived exosomes engineered with CMP could be internalized by H9C2 cells, an in vitro model for cardiomyocytes. Moreover, miR302 that was loaded into engineered exosomes was delivered with high efficiency to target cells, while DSPE-PEG-CMP-miR302-EXO significantly attenuated myocardial apoptosis and inflammatory response, as well as improved cardiac function. Our study demonstrates the potential of using engineered exosomes as vehicles for the targeted delivery of functional small RNAs for effective treatment of myocardial I/R injury.

## Data Availability

The original contributions presented in this study are included in the article/Supplementary material, further inquiries can be directed to the corresponding author.
